# Exploring critical intervention features and trial processes in the evaluation of sensory integration therapy for autistic children

**DOI:** 10.1186/s13063-024-07957-6

**Published:** 2024-02-17

**Authors:** Elizabeth Randell, Rachel McNamara, Monica Busse, Sue Delport, Rhys Williams-Thomas, Wakunyambo Maboshe, David Gillespie, Sarah Milosevic, Lucy Brookes-Howell, Melissa Wright, Richard P. Hastings, Anne Marie McKigney, Eleni Glarou, Alka Ahuja

**Affiliations:** 1 Centre for Trials Research, Cardiff University, Cardiff, UK; 2 School of Medicine, Cardiff University, Ty Dewi Sant, Heath Park, Cardiff, UK; 3 Centre for Educational Development, Appraisal, and Research (CEDAR), University of Warwick, Coventry, UK; 4 Aneurin Bevan University Health Board, Newport, UK; 5 Cardiff University, Cardiff, UK

**Keywords:** Process evaluation, Autism, Sensory integration, Manualised therapy

## Abstract

**Background:**

We evaluated the clinical and cost-effectiveness of manualised sensory integration therapy (SIT) for autistic children with sensory processing difficulties in a two-arm randomised controlled trial. Trial processes and contextual factors which may have affected intervention outcomes were explored within a nested process evaluation. This paper details the process evaluation methods and results. We also discuss implications for evaluation of individual level, tailored interventions in similar populations.

**Methods:**

The process evaluation was conducted in line with Medical Research Council guidance. Recruitment, demographics, retention, adherence, and adverse effects are reported using descriptive statistics. Fidelity of intervention delivery is reported according to the intervention scoring manual. Qualitative interviews with therapists and carers were undertaken to explore the acceptability of the intervention and trial processes. Qualitative interviews with carers explored potential contamination.

**Results:**

Recruitment, reach and retention within the trial met expected thresholds. One hundred thirty-eight children and carers were recruited (92% of those screened and 53.5% of those who expressed an interest) with 77.5% retained at 6 months and 69.9% at 12 months post-randomisation. The intervention was delivered with structural and process fidelity with the majority (78.3%) receiving a ‘sufficient dose’ of intervention. However, there was considerable individual variability in the receipt of sessions. Carers and therapists reported that trial processes were generally acceptable though logistical challenges such as appointment times, travel and COVID restrictions were frequent barriers to receiving the intervention. No adverse effects were reported.

**Conclusions:**

The process evaluation was highly valuable in identifying contextual factors that could impact the effectiveness of this individualised intervention. Rigorous evaluations of interventions for autistic children are important, especially given the limitations such as limited sample sizes and short-term follow-up as faced by previous research. One of the challenges lies in the variability of outcomes considered important by caregivers, as each autistic child faces unique challenges. It is crucial to consider the role of parents or other caregivers in facilitating access to these interventions and how this may impact effectiveness.

**Trial registration:**

This trial is registered as ISRCTN14716440. August 11, 2016.

**Supplementary Information:**

The online version contains supplementary material available at 10.1186/s13063-024-07957-6.

## Introduction

Individuals with sensory processing difficulties (SPD) experience disruption in the brain when processing information from sensory stimuli. SPD is a complex disorder which has been conceptualised as an ‘inefficient functioning of the central nervous system’ [1] and while it is not currently a recognised medical diagnosis in its own right, it is estimated to affect 90% of children on the autism spectrum [2–4] and has been included as diagnostic criteria for autism spectrum disorder (ASD) in the Diagnostic Manual for Mental Disorders-Fifth Edition (DSM-5) [5]. SPD often results in hypo- or hyper-reactive behavioural, motor or adaptive responses to external stimuli [6] though symptoms may vary widely depending on the sensory system affected and the severity of the difficulties experienced. Children may, therefore, require additional support to enable participation in routine activities in both home and school environments.

Occupational therapy (OT) interventions typically involve the use of various methods focused on improving occupational engagement, participation and performance. Some are labelled ‘top-down’ and focus on altering the child’s environment to accommodate their sensory processing difficulties. Others are thought of as ‘bottom-up’ as they concentrate on identifying and addressing the child’s difficulties in integrating sensory information and aim to facilitate adaptive responses to the environment. Sensory integration therapy (SIT) is a play-based therapy which takes the approach of addressing the child’s underlying neural difficulties. At the heart of SIT is a collaborative therapist–child relationship underpinned by key OT and fidelity principles to guide delivery. Therapy meeting these fidelity principles is trademarked as Ayres Sensory Integration™(ASI) [7]. ASI utilises individually tailored sensory-motor activities specific to each child with the aim of helping them improve their ability to process and integrate sensation [8]. Following a detailed assessment, therapists develop a hypothesis as to the nature of each child’s underlying sensory difficulty and help children and carers identify specific functional goals they want to achieve. The content of the therapy is then individualised to that child’s needs and undertaken in line with the ‘just-right’ challenge i.e. in a way that is appropriate for the child’s abilities. SIT meeting ASI criteria has been considered a potentially valuable therapy for children with SPD [9–11] however demonstration of clinical effectiveness had been lacking [12].

Randomised controlled trials of SIT are limited, with small sample sizes and lacking blinded evaluation [12]. Results have often been presented without clear protocols for delivery, assessment of fidelity or examination of contextual factors. All of these are critical elements of a complex intervention that should be explored through the inclusion of a process evaluation which ensures not only a better understanding of the theory of change of an intervention — but also helps to explain why specific components of an intervention might result in the intended outcomes [13]. Complex interventions are defined as having several interacting components with the complexity residing in the number and variability of these components [14]. The impact of context at any point in an intervention’s lifecycle, from conception through to implementation and evaluation, may then add a further layer to the complexity [15]. These types of interventions can be implemented across a wide range of settings and at different levels from a broad societal level down to a more specific individual level. Recent guidance on complex interventions proposes that research can adopt an ‘efficacy, effectiveness, theory based, and/or systems perspective’ depending on existing knowledge and the need for additional evidence to enhance understanding [13]. Evaluating a complex intervention efficiently may involve not only concentrating on a single effectiveness outcome but also gaining a comprehensive understanding of its broader impact across multiple domains. In this regard, inclusion of a process evaluation can provide valuable insights [16].

With SIT being accessed by parents of autistic children despite limited empirical support [12], a high-quality trial was needed to clarify its effectiveness and in July 2015, the National Institute for Health Research Health Technology Programme (NIHR HTA), put out a commissioning brief to address this. The brief emphasised demonstrating effectiveness through outcomes focussing on challenging behaviours; socialisation; engagement with activities; and sensory sensitivities. The resulting trial was the SenITA trial [17] — an effectiveness trial designed and implemented to answer the question of whether SIT has an impact on behavioural difficulties, adaptive skills and socialisation in children on the autism spectrum who have sensory processing difficulties. The primary outcome of interest was the level of behaviour problems as recorded at the 6-month timepoint. This outcome was measured using the Irritability subscale of the Aberrant Behavior Checklist (ABC-I) [18]. Currently, SIT, although described as a manualised intervention, is not associated with a formally defined programme theory to explain the way in which it is expected to work or a logic model to illustrate the theoretical underpinnings of the programme theory. Therefore, to complement the evaluation of the intervention, a process evaluation based on MRC guidance [13] was incorporated in the trial design. The anticipated benefit of this was to enhance understanding of the contextual factors influencing the delivery and uptake of the intervention.

Here we examine the results of the process evaluation and look at whether the intended population could be recruited into the trial and whether they stayed involved for the intended duration (‘reach’ and ‘retention’); whether the SIT intervention was delivered as intended (‘fidelity’); whether the amount of intervention delivered was as described in the trial protocol (‘adherence’); and the experiences of those who took part in the trial (‘acceptability’). We also examined any safety issues (‘adverse events’) and explored potential ‘contamination’ of SIT delivery within the trial – the extent to which participants allocated to the control arm receive treatment/therapy similar to those in the intervention arm.

## Methods and materials

### Trial processes

SenITA was a two-arm randomised controlled effectiveness trial (for detailed trial design see [17]). Children and their carers were recruited through specialist services (e.g. Child and Adolescent Mental Health Services (CAMHS), occupational therapy), primary schools, and via self-referral. The brief for the trial was specific in directing that the primary focus should be on behavioural outcomes, although the importance of assessing functional and carer outcomes was also highlighted. Irritability/agitation (as measured by the corresponding Aberrant Behavior Checklist subscale) [17], indicative of behaviour problems, at 6-month post-randomisation was chosen as the primary outcome. Secondary outcomes then included other problem behaviours, adaptive behaviours and functioning, socialisation, carer stress and quality of life.

Children were eligible to take part if they had a diagnosis of ASD OR probable/likely ASD (defined as currently being assessed within the local ASD pathway); were aged 4–11 years at the start of the trial; were in mainstream primary education; had SP difficulties [16]. Carers met with a researcher to go through the Patient Information Sheet in detail and to provide informed consent. Eligibility to take part was confirmed through a screening assessment. If eligible, participants then went on to complete baseline measures and were randomised to receive either the intervention (SIT) with usual care (UC) or to continue with just their UC. Throughout the trial, carers in both the intervention and usual care groups were asked to complete a diary to document any activities recommended by a professional for their child. Both groups also completed follow-up assessments at 6- and 12-month post-randomisation.

The trial ran between January 2017 and April 2021. Delivery of intervention sessions and follow-up assessments were impacted by restrictions in relation to the COVID-19 pandemic from March 2020 through to the end of data collection in October 2020.

### Intervention delivery

OT clinics were assessed to ensure they met full structural fidelity criteria for delivery of manualised SIT (Additional file 1) [7]. Prior to any intervention being delivered, two assessment sessions were completed to inform individualised intervention planning. Therapists assessed the child’s level of SPD using standardised measures, gained background history and an occupational profile from carers, and identified occupational goals using the Canadian Occupational Performance Measure (COPM) [19]. The 26-week intervention was then delivered in an intensive phase (two 1-h-long sessions a week for 10 weeks), a tapering phase (two sessions a month for 2 months) then one follow-up telephone call a month for 2 months. Other than the follow-up telephone calls, all sessions were delivered face-to-face.

### Process evaluation data collection

Components of the process evaluation were assessed and reported using mixed quantitative and qualitative methods. Using a convergent parallel design, mutually exclusive sets of data were created that informed each other. See Additional file 2 for a summary of data sources and the process evaluation elements they address.

#### Reach and retention

Baseline data were used to describe the characteristics of the recruited sample (e.g. age, degree of SPD and autism symptoms). Screening and baseline data were also collected to examine recruitment rates while completion rates of outcome data at 6- and 12-month follow-up were used to assess retention. To allow flexibility, assessments for all participants were scheduled within a ± 4-week window of each follow-up timepoint. Follow-up appointments could be rearranged twice for those who did not attend an arranged appointment.

#### Fidelity

Structural and process fidelity were scored and reported according to the Ayres Sensory Integration Intervention Fidelity Measure [7]. This measure demonstrates the degree to which the intervention could be consistently replicated and whether it meets the criteria of ASI. The first four parts of the measure address ‘structural fidelity’ and were assessed at the start of the trial only as they needed to be in place in order to conduct the intervention with the appropriate equipment and therapist training/mentoring. A minimum score of 85/110 was required to be part of the trial.

Parham et al. [7]. Ayres Sensory Integration Intervention Fidelity Measure. Used with permission.

To address process fidelity, intervention sessions were video recorded (provided participants consented) and delivery of the intervention was scored by qualified independent raters. A sample of intensive phase sessions was rated by at least one independent SIT-trained therapist (a randomly selected 15–20-min sample of the full session). To ensure consistency of ratings, a selection of sessions was rated by multiple independent SIT-trained therapists. An overall process score of at least 80 out of 100 (across 10 procedural elements, see Additional file 1.) was considered a demonstration of delivering the intervention in line with ASI principles. To demonstrate adequate fidelity, therapists were expected to score at least 80/100 on the process fidelity measure for at least 80% of sessions rated. Raters also gave a general impression of the intervention delivered by responding with ‘yes’ or ‘no’ to the statement ‘This intervention session is provided by a qualified therapist intentionally applying ASI intervention theory and methods’.

#### Adherence

Data recorded by 16 therapists delivering the intervention across eight sites/clinics were used to report attendance. Attending 13 of a possible 20 sessions delivered during the intensive intervention phase (two-thirds) was considered to be a ‘sufficient dose’. Clinical experience and available evidence [12, 20–22] have shown that change has been observed after this number of sessions.

#### Acceptability

Semi-structured interviews were conducted with 30 carers of children and 13 therapists involved in intervention delivery. Interviews examined their experience of the trial recruitment process, trial processes and measures, intervention implementation and acceptability, and contextual factors (see Additional files 3, 4, 5 and 6 for Topic Guides). Carers were purposively sampled ensuring representation of intervention and usual care participants, trial region and a mix of child sex and age. Before taking part in an interview, carers were asked to complete a template timeline of the support they and their child had received. This was used to facilitate discussion of their experiences. All therapists (*n* = 16) involved in delivery of the intervention were invited to take part in an interview.

#### Adverse events

The number of reported adverse effects was collected for children receiving the intervention. This was defined as any untoward medical occurrence in a participant who received the intervention which is not necessarily caused by or related to that intervention (as defined by Good Clinical Practice [23]). Adverse events that did not meet the criteria of serious were not systematically recorded. An adverse event would meet the criteria of serious if it resulted in death; was life-threatening; required hospitalisation or prolongation of existing hospitalisation; resulted in persistent or significant disability or incapacity; consisted of a congenital anomaly or birth defect; resulted in another medically important condition.

#### Contamination

Data to address contamination (i.e. whether participants assigned to the control arm received therapy consistent with SIT or received enhanced/additional support from clinicians aware of their involvement in the trial) was gained from parents and carers and compared to the expected provision mapped out in previous scoping focus groups [17]. Pre-recruitment, a brief survey of OTs and a series of focus groups/interviews with therapists and carers informed the definition of usual care (UC) [17].

### Data analysis

We described recruitment, demographics, retention, adherence, and adverse effects using frequencies with percentages, means with standard deviations and medians with interquartile ranges as appropriate. Fidelity ratings were reported according to the ASI scoring manual.

Qualitative interviews were audio recorded and transcribed verbatim. Anonymised transcripts were then analysed using a framework approach [24] by two qualitative researchers. First, key themes were identified which addressed the aims of the process evaluation; families' experience of support provided by services (carer interviews) and the perceived effectiveness of SIT (interviews with carers of children in the intervention arm and therapists). Then a second review of the transcripts was carried out by the first qualitative researcher to identify subthemes. Following a discussion of the subthemes with the second qualitative researcher, a thematic framework was created. All interviews were coded using NVivo v12 by the first qualitative researcher with 10% then coded by the second researcher. The two qualitative researchers discussed identified themes until consensus was reached.

## Results

Participants were recruited from August 2017 through to July 2019 (24 months). Follow-up ran from February 2018 through to January 2020. Recruitment was from five NHS Health Boards in South Wales, and two counties in England (Buckinghamshire and Cornwall). The results of the primary outcome showed no statistically significant effects of the intervention on the primary outcome of irritability/agitation at 6 months. There was also no evidence of meaningful intervention effects at 12 months across secondary measures of behavioural, adaptive functioning, socialisation, carer stress, health utility or quality-of-life measures [17].

### Reach

Of 258 carers who expressed an interest in taking part, 24 (9.3%) were not eligible while 84 went no further (no response *n* = 61; not interested in participating *n* = 23). Screening appointments were completed for the remaining 150 carers and children. Three children (2%) were ineligible after not meeting inclusion criteria and nine children did not progress (no response *n* = 4; not interested in participating *n* = 1; eligible and consented but withdrew immediately *n* = 4). This left a total of 138 children and carers who were randomised into the trial (100% of target sample size, 92% of those screened and 53.5% of those who expressed an interest in taking part). Based on their Sensory Processing Measure result at screening, 77.5% of those children recruited had scores indicative of ‘definitive dysfunction’. The majority were recruited from South Wales (71.7%). Children who took part had a mean age of 7.87 (SD 1.73) years; and the majority were male (79%), white British (88%) and in full-time mainstream school (77%). This is broadly representative of the wider population of children in primary education with a diagnosis of autism presenting to services [25] though we are aware that this isn't likely to be representative of the true population i.e. those who aren’t presenting to services. Children were mostly referred to the study by professional recommendation. Carers reported that they hoped their child would receive the intervention and that they wanted to take part to potentially improve support for autistic children with SPD in the future as they felt this was currently lacking and that their child had received little or no support previously.

### Retention

Retention reached 77.5% with 107/138 participants providing data at the primary outcome timepoint – 6 months post-randomisation. At this timepoint, 29 children/carers were lost to follow-up and two had withdrawn. One parent withdrew because completion of outcome measures created a reminder of the child’s challenging behaviours and the lack of support they were receiving for this. No reason was given for the other withdrawal. The final set of outcome data at 12 months was provided by 96 participants (69.6% of those recruited) with 40 lost to follow-up. There were no further withdrawals at this timepoint.

### Fidelity

All 16 therapists and their clinics met structural fidelity requirements for SIT with scores achieving the threshold of above 85/110 (range 96–110). Some clinics required the addition of specific equipment to achieve fidelity requirements, e.g. additional suspension points or equipment such as mats or swings. Scores were obtained for 12 different therapists delivering SIT to 46 different intervention participants across 96 sessions. Four independent raters provided 156 separate ratings for SIT delivered with a mean score of 90.1/100 (SD = 13.1). To ensure comparability among ratings, all four raters initially scored the same 17 sessions. In these cases, averages were taken of the four scores for each session. Some technical issues meant that recordings from four of the SIT therapists were not available to be rated.

Process fidelity scores showed that the intervention was delivered according to the underlying therapeutic principles on which it is based. Overall, 10 out of 12 therapists scored an average of at least 80/100 on process fidelity for at least 80% of their sessions. There was some variety in the scores, however. Therapist 7 failed to score an average of at least 80/100 for 50% of their sessions with the scores ranging from 49.25 to 77.75% (Table 1). Therapist 8 failed to score an average of at least 80/100 for 75% of their sessions (range 53–75.5% across 3 sessions). One rater consistently scored both Therapist 7 and 8 lower than the other raters which skewed their averages. Both therapists had mentors to support them further and improved in delivering the intervention. It was noted that while their clinic space met structural fidelity, it was also one that had limited flexibility. In addition, 92.3% of fidelity scores (144/156) also achieved a global impression score of ‘yes’, i.e. the intervention session was provided by a qualified therapist intentionally applying ASI intervention theory and methods.

**Table 1:** Fidelity scores

		Sessions averaging under 80/100		Sessions averaging over 80/100	
Therapist	Total number of sessions completed	Number of sessions	Range of average scores	Number of sessions	Range of average scores
1	16			16	85–97
2	34			34	82.5–100
3	14	1	57	13	84–100
4	2			2	93–99.25
5	4			4	85–100
6	2			2	88–100
7	8	4	49.25–77.75	4	85–94
8	4	3	53–75.75	1	95.5
9	2			2	84–97
10	2			2	97–100
11	6			6	91–100
12	2	1	79	1	91

### Adherence

A ‘sufficient dose’ of at least 13 SIT sessions was received by 78.3% of participants (54/69 of those allocated to the SIT arm) during the intensive stage. However, receipt of SIT sessions according to the protocol varied considerably between participants. Of 57 intervention participants who provided primary outcome data, 49 (86.0%) initiated SIT by 6 months and 38 (66.7%) received at least 13 SIT sessions in the intensive phase by that timepoint meaning 19 (33.3%) participants did not receive an adequate level of SIT. Pandemic-related reasons were indicated for 11 out of those 19 participants. In those who initiated SIT, the median time from randomisation to initiation was 48 (IQR 32–85) days. Across the whole intervention period, the median number of SIT sessions received was 20 (IQR: 16 to 21); the median number of SIT sessions received prior to primary outcome follow-up (in those with primary outcome data) was 18 (IQR 10–21).

### Acceptability

Data from qualitative interviews are presented here thematically under headings which reflect the frameworks created to address the aims of the process evaluation. Broadly, the overarching themes covered views on the research and views on the intervention. Within the research theme were sub-themes on overall experiences of the trial; trial information and appointments; completing trial measurements and assessments and randomisation. Within the intervention theme were sub-themes on intervention delivery; length of intervention and goal-setting; and potential for the adoption of SIT into usual care. A summary of the sub-themes is reported here, for a more detailed description of the data, please refer to the final report [17].

Quotes from carers are labelled with ‘P’ and a participant number; quotes from therapists are labelled with ‘T’.

#### Overall experiences of the trial

Overall experiences of trial participants were largely positive. Carers expressed their willingness to participate in the trial again if given the opportunity:

Oh definitely yeah, definitely. Without a shadow of a doubt, like I’d love to take part again, I know I can’t… honestly definitely it was totally worth it, I’d recommend it 100 percent. (P304, Intervention group)

Those with children in the intervention arm were happy that their child was receiving SIT and felt that their child’s difficulties were being acknowledged:

The OT was absolutely amazing. Like if I needed any advice on anything I could do with the certain situations, she gave me a solution. Again she suggested books and different activities I could do and that as well. (P109, Intervention group)

Carers felt that the trial was well-explained and did not feel pressured to participate. The reminder text messages, and flexible trial appointments were seen as beneficial. However, some reported a lack of communication from the trial team. For example, a carer in the control arm reported that they were not informed of which group their child was in and only realised their involvement in the trial months later when they received a reminder to fill in the diary. Another carer in the intervention arm reported that their child had not received therapy even after 6 months, and they were not informed about when it would start.

#### Trial information and appointments

Carers generally felt that they were provided with the right information about the trial before participating and had the opportunity to ask questions. Most carers felt well-informed and understood what would happen during the trial. However, there were some cases where carers felt they received too much information or that the information did not fully explain what would happen in the intervention sessions. Two carers were initially unaware that they had been allocated to the usual care group, causing confusion. Carers appreciated the time given to make a decision and felt that consent was checked at multiple stages.

Carers understandably reported being frustrated where there were delays in the delivery of SIT for various reasons. In some cases, this had been due to the COVID-19 pandemic though delays were also caused by everyday factors like therapist availability, sick leave, holiday etc. Therapists offered convenient and flexible appointment times where possible though this did not always match up with carer availability and school/working hours. Some schools were not consistently supportive of children attending SIT sessions during school time. There was also a degree of burden for some carers in terms of getting to sessions as only specific clinics (meeting fidelity criteria) were being used as part of the trial. For some, this meant a commitment to travel and additional time in their day to attend appointments.

It’s an hour and a half to the session… an hour and a half home, and that was twice a week. So it was a big financial investment to, you know, fund the fuel alone, and then with my time… It was a massive investment in family life… I would pick him up at 12 o’clock on the dot. He’d have lunch in the car, and we’d just about get to [the session] for 1.30. (P112, Intervention group)

#### Completing trial measures and assessments

Experiences of completing trial measures, including assessments and questionnaires, were mixed. Some carers found assessments to be positive and play-based, with good rapport between the assessor and child. However, there were challenges in conducting assessments, such as avoiding behaviours displayed by children and the length of the assessments. Some therapists had difficulty conducting assessments alone and required assistance from carers. Carers found the questionnaires quite long but easy to complete. Some felt the questions were useful in helping them reflect on their child’s needs while others felt they were upsetting to complete as they focussed on problematic areas of their child’s behaviour.

It was . . . really long winded . . . mentally and physically exhausting . . . It’s like when you go and do a [Disability Living Allowance] application . . . you’ve got to take your child at their worst ever possible day and write it down . . . you want to spend your life . . . focusing on what your child can [do], despite their difficulties, not what they can’t do, and I think it is a bit depressing like that. (P609, intervention group)

#### Randomisation

Carers generally had a good understanding of randomisation and appreciated the need for it, even if they were disappointed with their group assignment (all interviewees expressed a preference for the intervention arm). Those whose child was allocated to the control arm had been happy to continue being involved in the trial.

#### Intervention delivery

Therapists were engaged and positive when it came to delivery of the intervention. They enjoyed being able to spend longer with each child than they would typically as part of usual care. This meant they could develop an effective working relationship with children and carers. Carers were also positive about the intervention and appreciated how tailored it was to their child’s needs, interests and abilities.

I was absolutely staggered by the outcomes that we achieved. (T201)

[Sensory integration therapy is] really much more targeted and… it really felt as though we were getting to the bottom of and really kind of problem solving these… difficulties… with the child and with the family… I didn’t feel as though these children are going to need much more beyond this, whereas the usual care feels like, you kind of wonder when they’ll be back. (T210)

#### Length of intervention and goal-setting

Approximately half of the therapists felt the number of sessions being delivered were too many while others felt there were the right number and the intervention could even have been longer. Many carers felt that while the goal-setting approach to the intervention was positive, there were sometimes multiple issues they wanted to tackle and it was difficult to prioritise which would be best to focus on in the sessions. Therapists highlighted that carers often needed prompting to set more specific, functional goals.

#### Potential for the adoption of sensory integration therapy as usual care

Overall, carers felt SIT had a positive effect on their child and should be made available as usual care. Therapists were also in favour of providing SIT as usual care where appropriate and where resources were available. Some therapists felt that a sensory integration approach would be more useful alongside other approaches in achieving children’s goals.

I think that sensory integration therapy is just a tool in the occupational therapy toolbox . . . I don’t think it’s the first port of call. (T210)

### Adverse effects

During the trial, one Adverse Event was reported by a therapist. However, this was erroneously reported as it was clear after review that it did not meet the criteria of ‘serious’ as per the reporting guidelines and was not likely related to the intervention.

### Contamination of SIT intervention delivery within the trial

The treatment received by participants allocated to the usual care arm was sufficiently different to the intervention. This meant that treatment did not meet SIT fidelity criteria and that sessions were no more than once a week. However, one child allocated to usual care only had received intervention consistent with SIT; one started accessing support from local charities (not meeting fidelity requirements of SIT), and one had started a specialist school. Most therapists said their approach to delivering their usual care to children not in the trial had not changed as a result of their involvement in the trial. Often this was because they did not have the time to implement any of the sensory integration approaches in their usual practice.

## Discussion

The results of the process evaluation provide valuable insights into various aspects of the research, including participant recruitment, retention, fidelity of intervention delivery, adherence to the protocol, acceptability of the trial, and potential adverse effects. These findings inform priorities for future research and highlight key issues likely to impact any implementation of sensory integration therapy (SIT) for autistic children with sensory processing dysfunction (SPD). Process evaluation data offers insights into the results in the absence of a significant main effect of the intervention on the primary outcome.

Results show that participant recruitment was achievable; participants were recruited over a 24-month period from multiple sites; a significant proportion of carers and children who expressed interest in participating were ultimately randomised into the trial. Retention rates of 77.5% at the primary outcome timepoint and 69.6% at 12 months post-randomisation are satisfactory. While some participants were lost to follow-up or withdrew from the study, the overall retention rate was relatively high considering the length of the trial and the challenges associated with conducting this research during the outbreak of COVID-19. Qualitative insights offer potential reasons for attrition, such as completion burden (including the length of measures) and the sensitive nature of some of the carer-completed measures, where they focused on problematic areas of children’s behaviour. Understanding these factors can help improve retention strategies in future studies.

The intervention was delivered with fidelity to structural and procedural criteria and according to underlying therapeutic principles. The majority of therapists consistently scored above the threshold for process fidelity, although a few initially struggled to meet the criteria. These therapists received mentor support, which led to improvements in their delivery of the intervention. However, there was considerable variability in the receipt of sessions, with staff leave, sickness, holidays and pandemic-related reasons cited as contributing factors to inadequate delivery highlighting the potential for external factors to impact on treatment adherence in intervention studies of this nature.

Experiences of taking part were generally positive and feedback from carers and therapists indicated that there were no major issues in delivering the trial that for the most part, could not be resolved. Those in the intervention arm were particularly satisfied with their child receiving SIT and felt that their child’s difficulties were being acknowledged. However, some participants reported a lack of communication from the trial team, which could be addressed to enhance the overall trial experience. Contextual factors also include carers understanding of, and engagement with, the intervention; therapists’ therapeutic relationship with the child; and therapists support in being able to deliver the intervention. Additionally, logistical challenges, such as travel time and appointment scheduling, posed burdens for some carers, which could have impacted acceptability and engagement with the intervention. These challenges are particularly important to consider when thinking about barriers to children receiving an intervention since their access is dependent on the involvement of others, primarily parents or caregivers. Incorporating strategies to address these challenges may be useful for future trials, ensuring better accessibility and participation in the intervention.

No untoward safety concerns were reported as a result of the intervention, indicating the safety of SIT in this trial. However, there was an instance where an adverse event was incorrectly reported as a serious adverse event (SAE), highlighting the importance of accurate reporting and adherence to reporting guidelines. There was minimal contamination in the usual care arm as families allocated to SIT did not report receipt of any other significant intervention or contact with services.

Analysis of the primary outcome did not show SIT as having a significant impact on behaviour problems at 6 months (as measured by the ABC-I subscale), and although carers in the intervention arm reported high levels of satisfaction and benefit of SIT, there was no statistically significant change in carer stress or longer-term improvements in well-being and daily functioning following completion of therapy. Ultimately, the trial did not lead to significant changes in any measured outcome.

### Strengths and limitations

As described in the introduction, research into complex interventions ideally focuses on the theoretical underpinnings of an intervention and clarifying how it is understood to work. This often includes an examination of a logic model, and identifying what further evidence would be beneficial to the evidence base. A limitation of the SenITA intervention was there was no predefined programme theory; however, a strength of the study design was the embedded process evaluation which provided insights into the planning, delivery, and uptake of the intervention, and the contextual factors affecting the implementation, all of which could be used in future work on establishing a programme theory.

### Future considerations

Outputs from the process evaluation give an indication of the kind of considerations that might be beneficial for improving the way in which we conceptualise, describe, and evaluate the effectiveness of this type of individualised intervention. Individual-level tailored interventions recognise the unique needs and characteristics of each child. Evaluations would therefore benefit from considering how best to capture specific goals, preferences, and outcomes relevant to each individual. While core outcomes such as behavioural improvements, adaptive skills, and socialisation are commonly assessed, it is also important to capture the influence of contextual factors such as family support and practical challenges in intervention delivery. Understanding the role of these contextual factors is essential for interpreting and generalising the findings of evaluations across diverse populations and settings. Including the views of families, and clinicians in the evaluation process is crucial. Their perspectives and experiences can provide valuable insights into the effectiveness and acceptability of tailored interventions. This may be particularly important for interventions in populations who perhaps cannot voice their own needs or who rely on others to make decisions about their care. Trials in such populations may face similar difficulties because of the necessary involvement of others which adds to the complex nature of the intervention. These insights can be employed to form and shape a programme theory, offering valuable suggestions of the causal pathways through which the intervention is expected to act. This also applies to situations where a programme theory is already established and where additional refinement is deemed necessary.

## Conclusion

The SenITA process evaluation facilitated assessment of intervention delivery and the surrounding context. Despite the lack of effectiveness data on the intervention, the value of the process evaluation is to help identify contextual factors that may have had a bearing on this result. Understanding these contextual factors can help further define a programme theory for SIT. By considering the process evaluation alongside effectiveness outcomes in trials of individualised interventions, researchers can better understand the relationship between the intervention, the context in which it is delivered and its intended outcomes. This has the potential to play a critical role in adding to the evidence base for an intervention and guide the refinement and improvement of future interventions.

### Supplementary Information

**Additional file 1.** Ayres Sensory Integration™ Fidelity Measure^c^.

**Additional file 2.** Summary of data sources and the process evaluation objectives they address.

**Additional file 3.** SenITA Full Interview Topic Guide. Parents and Carers (SiT).

**Additional file 4.** SenITA Full Interview Topic Guide. Parents and Carers (Usual Care).

**Additional file 5.** SenITA Outline Interview Topic Guide. Therapists (Usual Care).

**Additional file 6.** SenITA Outline Interview Topic Guide. Therapists (SiT).

## Data Availability

Anonymised datasets during and/or analysed during the current study available from the corresponding author on reasonable request.

## Abbreviations

ABC-I: Aberrant Behaviour Checklist Irritability subscale
ASD: Autism Spectrum Disorder
ASI: Ayres Sensory Integration
COPM: Canadian Occupational Performance Measure
DSM-5: Diagnostic Manual for Mental Disorders-Fifth Edition
NHS: National Health Service
OT: Occupational therapy
RCT: Randomised controlled Trial
SAE: Serious Adverse Event
SBI: Sensory-Based Interventions
SIT: Sensory Integration Therapy
SPD: Sensory Processing Disorder
