# Supplementary material for: Exploring critical intervention features and trial processes in the evaluation of sensory integration therapy for autistic children
Source: Trials. 2024 Feb 17;25:131. doi: 10.1186/s13063-024-07957-6 (PMC10873975; doi:10.1186/s13063-024-07957-6)
Supplement: Supplementary file 1 — Additional file 1. Ayres Sensory Integration™ Fidelity Measurec. [file 13063_2024_7957_MOESM1_ESM.docx]

**Additional File 1. Ayres Sensory Integration**™ **Fidelity Measure^c^**

| **Structural fidelity** |  |
| --- | --- |
| Therapist qualifications (including professional training and mentorship) | /10 |
| Record review | /38 |
| Room specification and equipment | /56 |
| Parent–therapist collaboration on goal setting | /6 |
| Total | /110 |
| **Process fidelity** |  |
| The therapist ensures physical safety by attending to the child's abilities and potential dangers. | /10 |
| The therapist presents the child with at least two of the following three types of sensory opportunities: a) tactile, b) vestibular, c) proprioceptive. | /10 |
| The therapist supports sensory modulation for attaining/maintaining a regulated state including arousal, alertness, affect, and activity level. | /10 |
| The therapist challenges postural, ocular, oral and/or bilateral motor control. | /10 |
| The therapist challenges the child's praxis and organization of behavior ability including the ability to conceptualize and plan novel motor tasks, and organize his or her own behavior in time and space. | /10 |
| The therapist collaborates in activity choice with the child. Activity choices and sequences are not determined solely by the therapist. | /10 |
| The therapist tailors activity to present just-right challenge and suggests or supports an increase in complexity of challenge when child responds successfully. | /10 |
| The therapist ensures that activities are successful by facilitating challenges in which the child can be successful in making an adaptive response. | /10 |
| The therapist supports child's intrinsic motivation to play and creates a setting that supports play as a way to fully engage the child in the intervention. | /10 |
| The therapist establishes a therapeutic alliance that promotes and establishes a connection with the child, working together toward one or more goals in a mutually enjoyable partnership. | /10 |
| Total | /100 |
