# Supplementary material for: Exploring critical intervention features and trial processes in the evaluation of sensory integration therapy for autistic children
Source: Trials. 2024 Feb 17;25:131. doi: 10.1186/s13063-024-07957-6 (PMC10873975; doi:10.1186/s13063-024-07957-6)
Supplement: Supplementary file 2 — Additional file 2. Summary of data sources and the process evaluation objectives they address. [file 13063_2024_7957_MOESM2_ESM.docx]

**Additional File 2.**

**Summary of data sources and the process evaluation objectives they address**

| Data source | Addresses recruitment, adherence, reach | Addresses intervention fidelity | Addresses intervention acceptability | Addresses implementation of SIT within an RCT | Addresses feasibility of data collection |
| --- | --- | --- | --- | --- | --- |
| Baseline trial data on sample characteristics | ✓ |  |  |  |  |
| Intervention attendance data | ✓ |  |  |  |  |
| Video-recordings of intervention sessions |  | ✓ |  |  |  |
| Baseline and follow-up outcome data |  |  |  |  | ✓ |
| Interviews with therapists | ✓ | ✓ | ✓ | ✓ |  |
| Interviews with parents/carers | ✓ | ✓ | ✓ | ✓ |  |
