# Supplementary material for: Exploring critical intervention features and trial processes in the evaluation of sensory integration therapy for autistic children
Source: Trials. 2024 Feb 17;25:131. doi: 10.1186/s13063-024-07957-6 (PMC10873975; doi:10.1186/s13063-024-07957-6)
Supplement: Supplementary file 3 — Additional file 3. SenITA Full Interview Topic Guide. Parents and Carers (SiT). [file 13063_2024_7957_MOESM3_ESM.docx]

**SenITA Full Interview Topic Guide**

**Parents and Carers (SiT)**

**Timeline discussion**

- (If completed) Could you talk me through the timeline?
- Could you tell me a bit about your child’s sensory problems?
- Are there any key events in the past that you can think of that have led to a change in your child’s behaviour?
- Have there been any key events or significant people involved in the support you and your child have had for their autism?
- Could you tell me about the therapies or interventions your child has had for his/her autism? (e.g. Speech & Language Therapist, Occupational Therapist, Paediatrician, Psychologist, Counsellor, Dietician, Teacher)
  - Have any of the therapies or interventions been particularly useful?
  - Have any not been useful?
- Could you tell me about any strategies, ideas or advice you have been given in relation to your child’s autism? For example, this could be from education or health professionals, local or national support groups, family or other parents.
  - Have any of these strategies or ideas been particularly useful?
  - Have any not been useful?
- Are there any therapies, interventions or strategies that you tried for a very short time and then stopped? What were the reasons?
- Are there any particular therapies, interventions or strategies that you have tried that you would recommend to someone else?
- Have you encouraged your child to participate in any activities or groups to help them?

**Experience of the intervention**

- Could you tell me about your and your child’s overall experience of the sensory integration therapy intervention?
  - Has it been a positive or a negative experience overall?
  - How has your child reacted to the intervention?
  - Are there any ways the intervention has been particularly good or bad? For you? For your child?
  - Have you noticed any change in your child’s behaviour since starting the intervention? (If any improvement) Has this lasted?
  - Has anyone (such as family members, school staff or other professionals) commented on a difference in your child?
  - Would you recommend the intervention to other parents?
- Has the intervention provided you with any activities or strategies to use at home?
  - Have you tried any of these ideas?
  - How successful has this been?
  - Has there been anything that has stopped you using particular ideas or strategies from the intervention?
- How easy or difficult was it to come up with goals at the beginning of the intervention?
- Was there any point part way through having the intervention sessions where you felt there had been a change in your child?
- In what ways has the intervention been different from the usual support you and your child receive?
- What other groups, support or activities have you or your child participated in while the intervention has been going on?
  - Have you or your child started any new groups, support or activities since starting the intervention?
- How easy or difficult was it for you and your child to take part in the intervention?
  - What made it easy/difficult?
  - How easily did the intervention fit in with family life and work or school?
  - Were you able to attend all of the intervention appointments?
  - Were the appointments convenient? (time / location)
- Can you think of any ways in which the intervention could be improved?

**Taking part in the SenITA study**

- How did you find out about the SenITA study?
  - How was it explained to you?
  - Was the information you were given about the study easy to understand?
  - Did you have any concerns about taking part in the study?
  - Has taking part in the study been what you expected?
  - Do you feel that you were given the right information about the study before you took part? Is there anything you were not told that you would have liked to have known?
- What made you decide to take part?
- Did you know that there were two groups of children, one group chosen at random that would receive the intervention and one that wouldn’t?
  - How did you feel about the process of randomly selecting children for each group?
  - Were you happy that your child was selected to receive the intervention?
  - Would you have been happy if your child had been selected not to receive the intervention?
- What did taking part in the SenITA study involve for you altogether?
  - Did you complete a diary, online or on paper?
  - Did you have any difficulty completing this? Was it clear what you had to do?
  - Did you complete questionnaires?
  - How easy or difficult did you find these to complete? Were there any questions that were not clear?
- Has taking part in the study taken more or less of your time than you thought?
- If you went back in time, would you take part in the study again?

_________________________________________________________________________________

**End of interview**

- We’ve covered all of my questions – is there anything we haven’t mentioned that you’d like to talk about?
- Thank you for taking the time to talk to me today
