# Supplementary material for: Exploring critical intervention features and trial processes in the evaluation of sensory integration therapy for autistic children
Source: Trials. 2024 Feb 17;25:131. doi: 10.1186/s13063-024-07957-6 (PMC10873975; doi:10.1186/s13063-024-07957-6)
Supplement: Supplementary file 5 — Additional file 5. SenITA Outline Interview Topic Guide. Therapists (Usual Care). [file 13063_2024_7957_MOESM5_ESM.docx]

**SenITA Outline Interview Topic Guide**

**Therapists (Usual Care)**

- Experience of taking part in the study
- Did support delivered differ as a result of the study?
- Allocated time: time allocated for preparation and support; expectations vs. reality
- Study recruitment: achieving recruitment targets; challenges/barriers to recruitment and how to overcome these
- Mentoring: experience and process of being a mentor/mentee
- Additional support needed: e.g. peers, checking, etc.
- Any factors (not pre-empted) that affect the ‘quality’ or type of support they are able to give children
- Any change in these factors over time (e.g. beginning vs. end of study, once therapists have seen more children, etc.)
