# Supplementary material for: Exploring critical intervention features and trial processes in the evaluation of sensory integration therapy for autistic children
Source: Trials. 2024 Feb 17;25:131. doi: 10.1186/s13063-024-07957-6 (PMC10873975; doi:10.1186/s13063-024-07957-6)
Supplement: Supplementary file 6 — Additional file 6. SenITA Outline Interview Topic Guide. Therapists (SiT). [file 13063_2024_7957_MOESM6_ESM.docx]

**SenITA Outline Interview Topic Guide**

**Therapists (SiT)**

**Intervention delivery**

- Please could you start by telling me about your experience of delivering the sensory integration therapy intervention?
- How many children have you delivered the intervention to?
- Has it been a positive or a negative experience overall?
- How easy or difficult did you find it to deliver the intervention?
  - Was there anything that made delivering the intervention problematic or difficult?
  - Was there anything that helped you or that you found useful in delivering the intervention?
  - Were there any unexpected factors that affected the quality or type of intervention or support you were able to provide? If yes, was there any change in these factors over time? (e.g. beginning vs. end of the study; once you had seen more children, etc.)
  - Do you feel sufficient time was allocated for you to deliver the intervention, including preparation time?
  - Did you need any additional support to deliver the intervention?
- Overall, what do you think worked well about the intervention?
  - Was there anything that didn’t work so well?
- Did you have to adapt the intervention in any way?
  - For particular children? (e.g. according to ability/motivation and/or type of sensory difficulty)
  - To suit your local context?
  - Did you have to change your style of delivering therapy?
- Is there a point in the sessions where you feel there is a consistent change in children? (e.g. at the halfway point)
- Have you had more than one child at once on your caseload receiving the intervention?
  - If yes, how did this work for you? Were the children in different phases of the intervention?
  - If no, do you think you would have been able to do this?
- How did you find working with the equipment?
  - How much time has it taken to set up the room before each session?
  - How does this compare with the time you would take to set up the room before a usual session?
- How did you find doing the assessments?
  - Do you feel you had long enough to complete the assessment?
- How did you find parents and children reacted to coming up with goals? Did they require a lot of prompting?
- How did you find the transition between the intense phase of the intervention where you were seeing the child every week, and the subsequent phases where you had less frequent contact?
- Have you discussed the intervention with colleagues who are not taking part in the SenITA study? Do you know if they have used any of the ideas in their practice?

**Intervention vs. usual care**

- How was the intervention different to the support you would normally provide?
- What do you feel was the effect of the intervention on the children you worked with?
  - Do you think the intervention made a difference?
  - As far as you know, has it had an impact on children’s behaviour?
  - What was children’s reaction to the intervention?
- What has been parents’ reaction to the intervention?
  - Do you think parents have found the intervention useful?
  - Have parents been receptive to suggestions of activities or strategies?
  - Do you think the intervention has had an impact on parental stress and quality of life?
- Do you think that either usual care or the intervention is a better option for children?
- Do you think the intervention should be adopted as usual care?
  - For all children? Certain groups of children? (e.g. those of a particular age/ability or with a particular type of sensory difficulty)
  - What do you think the impact would be on you and your colleagues if the intervention was adopted as usual care?
  - Are there any changes that would need to take place to be able to deliver the intervention as standard care in the future?
  - Can you foresee any potential issues or barriers?
- Do you think your approach to delivering usual care to children not receiving the intervention has changed as a result of you being part of the study?

**SenITA study processes**

- Have you had any involvement in identifying potential participants for the SenITA study?
  - How did you go about this?
  - Were there any particular challenges? If yes, how did you overcome these?
- Were you involved in explaining the study or intervention to participants?
  - How easy or difficult was it to explain the study to parents? To children?
  - How easy or difficult was it to explain the intervention to parents? To children?
  - Did parents understand the study information?
  - What has parents’ reaction been to the study?
  - Did they have any particular concerns?
- How easy or difficult was it to stick to the study protocol or manual?
  - Do you think any parts of the study protocol or manual are too rigid or could be made more flexible?
  - Were you able to stick to study procedures? Did you have to make any changes?
- How did you find completing all the measures or forms required for the study?
  - Were there any measures that were confusing or difficult to complete?
  - Do you feel you had sufficient training and support to complete study measures?
  - How did you find using the iPad?
  - Did you have any issues recording or uploading data?
  - Did you have any problems using the iPad to look up data?
  - Did you have any issues videoing the sessions?
- Did your expectations of what would be required of you match the reality of being involved in the study?
  - Was it more or less work than you’d thought?
- Were there any aspects of the way the study was designed or run that you thought worked well?
- Were there any aspects of the study that you thought were problematic or did not work well?

**Support and training**

- How prepared did you feel for delivering the intervention?
  - Do you feel you had sufficient training in intervention delivery? (If no, what was missing/ how could the training have been improved?)
  - Do you feel you had sufficient support in delivering the intervention?
- How prepared did you feel for taking part in the study?
  - Do you feel you had sufficient training on the study? (If no, what was missing/ how could the training have been improved?)
  - Do you feel you had sufficient support while taking part in the study?
- Was there anything you think it would have been useful for you to have known before taking part in the study?
- How useful did you find the manual?
- Did you find completing the reflective questions useful?
- Have you used the study Facebook group?
  - If no, why not?
  - If yes, has this been useful? How have you used it?
- Please could you tell me a bit about your experience of being mentored as part of the study?
  - How much mentoring did you have?
  - How useful did you find the mentoring sessions?
  - Did you experience any difficulties sharing data or session videos with your mentor or mentors?

__________________________________________________________________________________

**End of interview**

- We’ve covered all of my questions – is there anything we haven’t mentioned that you’d like to talk about?
- Thank you for taking the time to talk to me today
